# Alerted default mode: functional connectivity changes in the aftermath of social stress

**DOI:** 10.1038/srep40180

**Published:** 2017-01-05

**Authors:** Benjamin Clemens, Lisa Wagels, Magdalena Bauchmüller, Rene Bergs, Ute Habel, Nils Kohn

**Affiliations:** 1 Department of Psychiatry, Psychotherapy and Psychosomatics, Medical School, RWTH Aachen University, Pauwelsstrasse 30, 52074 Aachen, Germany; 2 Jülich Aachen Research Alliance (JARA) – Translational Brain Medicine, Aachen, Germany; 3 Institute of Neuroscience and Medicine, Computational and Systems Neuroscience (INM-6), Wilhelm-Johnen-Straße, 52425 Jülich, Germany; 4 Radboudumc, Donders Institute for Brain, Cognition and Behaviour, Department for Cognitive Neuroscience, Kapittelweg 29, 6525 EN Nijmegen, The Netherlands

## Abstract

Stress affects the brain at a network level: the salience network is supposedly upregulated, while at the same time the executive control network is downregulated. While theoretically described, the effects in the aftermath of stress have thus far not been tested empirically. Here, we compared for the first time resting-state functional connectivity in a large sample of healthy volunteers before and after a mild social stressor. Following the theoretical prediction, we focused on connectivity of the salience network (SN), the executive control network (ECN) and the default mode network (DMN). The DMN exhibited increased resting-state functional connectivity following the cyberball task to the key nodes of the SN, namely the dorsal anterior cingulate cortex (dACC) and the anterior insula, as well as sensorimotor regions and higher-order visual areas. We conclude that this increased connectivity of the DMN with key nodes of the SN and regions responsible for preparatory motor activity and visual motion processing indicates a shift towards an ‘alerted default mode’ in the aftermath of stress. This brain response may be triggered or aggravated by (social) stress induced by the cyberball task, enabling individuals to better reorient attention, detect salient external stimuli, and deal with the emotional and affective consequences of stress.

Stressful experiences strongly influence human behavior and underlying neural processing. While brain responses to acute stress have been investigated within several experimental paradigms including social or physiological stressors, fewer studies focused on the prolonged impact of stress. A recent model based on temporal and spatial specificity of stress related neuromodulators proposed dynamics of large-scale brain networks (namely salience and executive control) that underlie an adaptive stress response^1^. So far only a few studies examined how connectivity within different large-scale brain networks (measured by i.a. resting state functional connectivity) might be modulated by stress. Following experimentally induced psychological stress, previous fMRI studies found increased resting-state functional connectivity of the amygdala with brain areas such as the hippocampus, medial prefrontal cortical structures, and the anterior insula^2^,^3^,^4^. In response to stress induced by Pavlovian fear conditioning, increased resting-state functional connectivity was observed between the amygdala and the dorsal prefrontal cortex (PFC)^5^. Prolonged stress exposure in medical students i.e. leads to functional changes in several large-scale brain networks^6^,^7^. High stress as induced via presentation of highly aversive visual stimuli combined with a pharmacological intervention (β-adrenergic receptor blockade via propranolol) results in profound changes of interconnectivity within the salience network (SN). These changes seem to be mediated by noradrenergic bottom-up processes supporting the conceptual link of neurochemical processes and a large-scale brain network balance^1^.

In detail the neural stress process can be defined by selective spatial and temporal effects initiated by catecholamines and glucocorticoids. Early and fast catechiolaminergic stress responses upregulate the SN. The SN usually includes the amygdala, the anterior middle cingulate (aMCC; dorsal anterior cingulate, dACC), anterior insula, thalamus, temporo-parietal cortices, striatum and the brainstem^8^,^9^,^10^,^11^. Altered connectivity within the SN is paralleled by changes in the executive control network (ECN). Simultaneously, the ECN, which encompasses the dorsolateral prefrontal cortex, dorso-medial prefrontal cortex and dorsal posterior parietal cortex^1^,^12^, is suppressed in acute stress and up-regulated in the aftermath of stress. In the aftermath of stress (>1 hour) slower effects of glucocorticoids initiate a down-regulation of SN and also ECN connectivity. Additionally, maladaptive reallocation of neural resources, also driven by noradrenergic bottom-up modulation triggered by the stressor is associated to default mode network hyperactivity^1^,^13^. Notably, this theoretical framework primarily provides a basis for neurochemical processes elicited during and after acute stress. So far, it has not been applied to social stressors, which strongly depend on individual evaluations.

In general, humans are highly motivated to be included by others. Therefore, exclusion in a social context is a likely source of stressful experiences^14^,^15^,^16^. Modulated by inta-individual (genetic, motivational) or contextual factors, social exclusion represents a ‘relative’ stressor rather than an ‘absolute’ stressor^17^. Nevertheless, social evaluative stress, as evident during social exclusion constitutes a psychological stressor^18^ as it reliably threatens basic social needs. Extensively studied, the most common and ecologically valid experimental task for measuring social exclusion – and the associated stress – is the cyberball task^19^,^20^. Exclusion periods within this virtual ball tossing game, threaten the need to control and belong resulting in negative affect and increased stress^15^,^19^,^21^. FMRI studies employing the cyberball task revealed a distributed neural network associated with social stress. This network comprises the dorsal anterior cingulate cortex (dACC), subgenual ACC (sgACC), OFC, lateral PFC, and the insula^15^,^22^,^23^,^24^.

The primary goal of the present study was to compare large-scale brain networks associated to adaptive stress responses before and after participants completed the cyberball task. With this exploratory study, we want to determine the influence of social stress on large-scale brain networks in a large sample of healthy participants. With respect to previous findings, our main focus was to determine whether social stress modulates large-scale brain networks comparable to non-social stress induction and to theoretical models.

For the present study, we hypothesized that social stress induced by social exclusion might significantly influence the dynamics of large-scale brain networks that have previously been associated to stress. More specifically, we hypothesized an altered response to social stress induced by social exclusion within the SN and the ECN^1^,^8^. In line with previous results on differential reallocation of resources in stress^13^, current developments of the brain stress model^25^ and as the DMN is intrinsically related to both SN and ECN, we included the DMN in our study. Consequently, the DMN, executive control and SN were of primary interest for the present study.

## Materials and Methods

### Participants

All participants recruited for the present study were healthy adult volunteers. Public announcements were used to recruit participants from Aachen (Germany) and the region. We verified that all participants had no contraindications against MR measurements, normal or corrected vision, and no history of traumatic brain injury, psychiatric or neurological illness. Using the Edinburgh Handedness Inventory (Oldfield, 1971), all participants were classified as fully right handed. Overall, 89 healthy volunteers (mean age = 26 years; SD = 8 years) were included in the present study, comprising 43 female and 46 male participants. The authors assert that all procedures contributing to this work comply with the ethical standards of the relevant national committee on human experimentation (i.e., Ethics Committee of the Medical Faculty of the RWTH Aachen University) and with the Helsinki Declaration of 1975, as revised in 2008. All experimental procedures and protocols contributing to this work were approved by the Ethics Committee of the Medical Faculty of the RWTH Aachen University (EK 291/12). Furthermore, all participants gave their written informed consent and received compensatory payment for participation.

### Cyberball task

During the cyberball task, participants believe that they play a virtual ball tossing game with two other participants to whom they are supposedly connected via a computer. In reality, the actions of the other two players are pre-defined and participants can be either included or, after a few catches, excluded from the ball tossing game. For the present study, we employed an adapted, MR suitable version of the cyberball task, which was originally developed by Williams^19^. Please note that data from a sub-group of the whole sample is subject to another separate publication, which does not focus on resting-state analyses but examines the neural and behavioral effects of the cyberball task itself^26^.

Upon arrival at the scanner, participants were informed that they take part in a group study, which was designed to test their mental visualization skills while they were playing a virtual ball tossing game on a computer. Participants were told that their computer would be connected to the computer of two other players sitting in adjacent rooms. During the game, the opponents were represented by two virtual players located on the right and left side of the screen, called “Dieter” and “Nora,” with their respective names written above graphic illustrations of the figures. “Dieter” always started the game by throwing the ball to the participant, who was represented by a hand in the lower center of the screen. Details on the procedure are reported elsewhere^26^. After each inclusion and exclusion block, the participants rated their subjectively felt affective state (valence) and (for a subset only) their subjectively felt anger on a 9-point scale. Additionally, before and after the cyberball task participants completed the Positive and Negative Affect Schedule (PANAS), to assess changes in affective state. The PANAS has been used previously to successfully assess affect changes^27^,^28^.

### Behavioral exclusion effects

We used SPSS (IBM Corp. Released 2012. IBM SPSS Statistics for Windows, Version 21.0. Armonk, NY: IBM Corp.) in all behavioral analyses. For all parametric test assumptions were not violated, all t-test were two-tailed, and p-thresholds for significance testing were set to 0.05.

Two paired t-tests for affective state and anger after inclusion versus exclusion periods were conducted to determine the exclusion effect.

### Mood change

We additionally tested significant changes in the mood state in a 2 × 2 repeated measures ANOVA (pre versus post, and positive versus negative PANAS values).

### Integration of brain and behavior

We wanted to assess if change in connectivity values were related to the behavioral effects. Therefore for the exclusion effect, we entered mean connectivity scores (for dACC and IFG clusters only, difference score post minus pre, see description below) as covariates in an analogue repeated measures ANOVA (exclusion versus inclusion as repeated measures). For the mood change analyses we added the same covariates to the 2 × 2 repeated measures ANOVA described above.

### Image acquisition

A Siemens 3T Trio scanner (Siemens AG; Erlangen, Germany), located at the RWTH Aachen University Hospital, equipped with a 12-channel head matrix coil was used for fMRI measurements. For stabilization of the head during scanning, foam pads were used. Overall, there were three functional runs and one anatomical run for each participant. The first run comprised a resting-state measurement, which was 6 min and 40 s long, followed by the cyberball task, which lasted approximately 24 min. The third run comprised the second resting-state measurement, followed by the anatomical scan (approximately 10 min). The total measurement time for each participant was approximately 50 min. During resting-state measurements, participants saw a black screen and were instructed to keep their eyes open without falling asleep. 250 functional images were acquired for each resting-state run, using a spin-echo EPI sequence with the following acquisition parameters: TR = 1600 ms, TE = 30 ms, flip angle = 67°, FOV = 192 × 192 mm^2^, matrix size = 64 × 64, 26 transversal slices, voxel size = 3 × 3 × 4.2 mm^3^. The anatomical scan was employed to acquire high-resolution anatomical images for each participant, using an MPRAGE sequence with the following acquisition parameters: TR = 2300 ms, TE = 3.03 ms, flip angle = 9°, FOV = 256 × 256 mm^2^, 176 sagittal slices, voxel size = 1 × 1 × 1 mm^3^.

### Image processing and analyses

FSL (FMRIB, University of Oxford, UK; www.fmrib.ox.ac.uk/fsl;^29^) was used for pre-processing, data-denoising, dual regression, as well as group analyses. For pre-processing, the first 5 volumes of each functional time series were discarded, allowing the brain to reach a stable magnetized state and preventing artifacts from transient signal changes at the beginning of functional runs. Other pre-processing steps included three-dimensional movement correction, spatial smoothing using a 6 mm full-width at half maximum (FWHM) Gaussian kernel to reduce inter-subject variability, and a high-pass filter (>0.01 Hz). All pre-processing steps except for temporal filtering were conducted before data denoising using ICA-AROMA^30^,^31^. By matching single subject ICA components to four robust and standardized features, ICA-AROMA identifies and removes motion related artifacts, which otherwise might induce spurious temporal correlations between brain areas. Data denoising is conducted by linear regression of ICA components identified as noise by AROMA. Finally, data were normalized to MNI space and re-sampled to 2 mm^3^ resolution using FMRIB’s Nonlinear Image Registration Tool (FNIRT) implemented in FSL.

Preprocessed and denoised resting-state data were analyzed using the dual regression (DR) technique, allowing for voxel-wise comparisons of functional connectivity at rest^32^.

For the present study, the templates for these three large-scale brain networks were taken from studies that provide representative findings regarding the specific networks: templates for the ECN and the DMN were taken from the study by Smith and colleagues^12^, whereas the template for the SN was taken from the study by Shirer and colleagues^11^. Unthresholded z-maps of these networks were temporally concatenated in one 4D file and used as input for the dual regressionin a linear model fit against the fMRI data, resulting in the subject-specific temporal dynamics for the templates. Subsequently, these temporal dynamics, or time-course matrices, are employed in a linear model fit against the associated fMRI data set to estimate subject-specific spatial maps. The different spatial maps for all participants and scanning sessions are combined into a single 4D file per ICA component. From these t-map components, difference scores for pre-cyberball minus post-cyberball and vice versa were calculated. The spatial maps resulting from this last step were modeled to characterize differences between the two scanning sessions, enabling us to examine differences between pre- and post-cyberball large-scale brain networks. Thresholding and correction for multiple comparisons within a volume for these spatial maps was achieved using the threshold-free cluster enhancement (TFCE) method^33^, resulting in a whole-brain corrected significant threshold of *p* < 0.05and employing nonparametric permutation testing with 5000 permutations^34^. Finally, statistical maps were superimposed on the MNI 152 template brain provided in MRIcro GL^35^ (http://www.mccauslandcenter.sc.edu/mricrogl/).

In a post analysis step, data from the t-map difference scores was extracted per subject using the command fslmeants implemented in FSL. For this we generated a mask including all significant voxels from the dual regression analyses and calculated the mean value of all voxels per cluster. This gives us an indication of mean connectivity change within one cluster per subject.

## Results

No significant differences between the first and the second resting-state measurement were found in SN and ECN. For the DMN, we found differences between pre- and post-cyberball measurements. In the contrast post-cyberball > pre-cyberball, significant differences in resting-state functional connectivity were found in the following regions, which all exhibited increased connectivity with the DMN after the cyberball task. Two clusters were located in the occipital cortex, with the first cluster covering the calcarine gyrus and the medial aspects of the secondary visual cortex (V2). The second occipital cluster was located close to the intersection of the transverse occipital and the intraparietal sulci, covering the higher-order visual area V3a. These two visual areas spanned both Brodmann areas (BA) 18 and 19. Furthermore, two clusters were located in the vicinity of the central sulcus of the right hemisphere, with the first cluster covering the premotor cortex, or pre-supplementary motor area (pre-SMA), at BA 6, involving parts of the superior and the middle frontal gyrus (MFG). The second cluster covered the primary somatosensory cortex at the post-central gyrus, involving BA 3.

**Figure 1:**
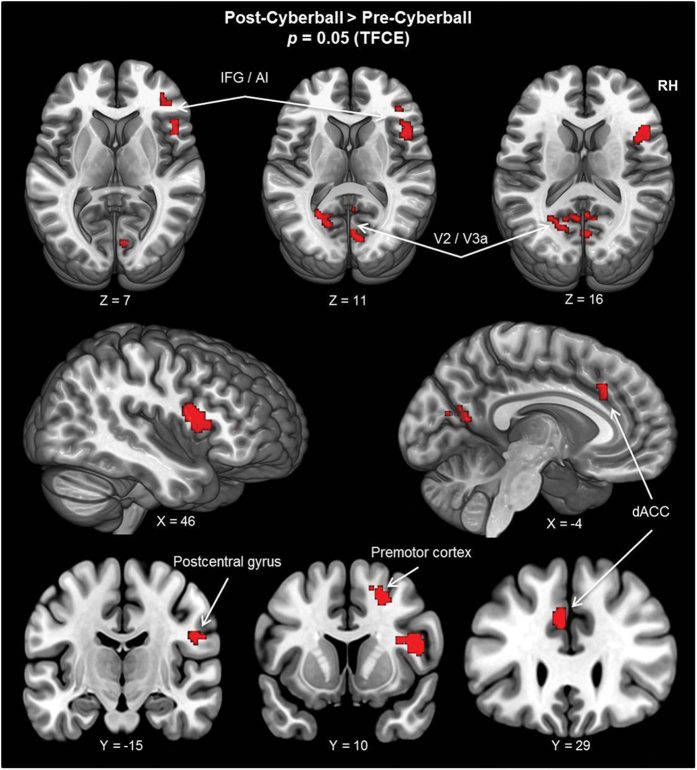
For the DMN, changes in resting-state functional connectivity between the two resting-state measurements are projected on the MNI template brain (ICBM 152). Shown are all areas which exhibited increased functional connectivity with the DMN following the social stress-inducing cyberball task (i.e., post-cyberball > pre-cyberball). X, Y, and Z coordinates refer to MNI coordinates, indicating which slice is depicted. Thresholding and correction of multiple comparisons was achieved using the threshold-free cluster enhancement (TFCE) method, resulting in a whole-brain significant TFCE threshold of *p* < 0.05. (*AI* = *anterior insula; dACC* = *dorsal anterior cingulate cortex; DMN* = *default mode network; RH* = *right hemisphere; IFG* = *inferior frontal gyrus*).

Another cluster was located at the dACC, covering BA 32. Whereas this cluster comprised a primarily medial region of the brain (dACC), its peak coordinate was located within the left hemisphere. Finally, two clusters were located in the vicinity of the right inferior frontal gyrus (IFG), both covering the anterior part of the insula. Of these two clusters, the first spanned the IFG and the anterior part of the insula at BA 44, whereas the second cluster was located at the intersection of BA 45 and 47, covering parts of the IFG, MFG and anterior insula. All results are illustrated in Fig. 1, and peak coordinates and other relevant cluster information are summarized in Table 1. The contrast pre-cyberball > post-cyberball did not reveal any changes in resting-state functional connectivity for the DMN.

### Behavioral exclusion effects

**Figure 2:**
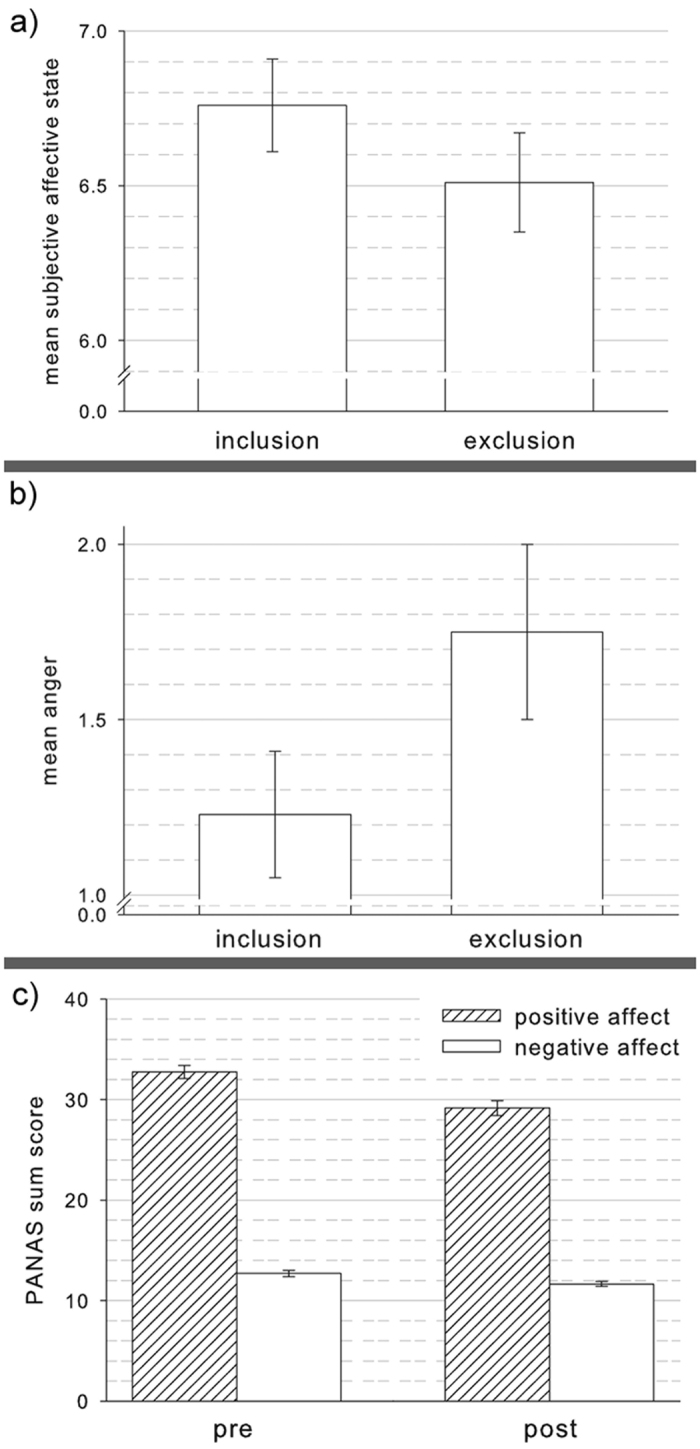
Depicted are the mean scores for (**a**) subjective affective state (valence) of 88 participants, (**b**) anger of 49 participants and (**c**) positive affect (PA) and negative affect (NA) derived from the Positive and Negative Affect Schedule (PANAS) of 88 participants. Graphs (**a**) and (**b**) depict mean values for exclusion and inclusion periods during the cyberball and (**c**) depicts mean values before (pre) and after (post) the complete cyberball task. In (**a**) and (**b**) the difference between exclusion and inclusion was significant for both, in (**c**) we found pre differed significantly from post, positive from negative affect and the interaction between pre-post and post-negative affect was also significant, driven by a more pronounced decrease in positive affect compared to negative affect.

We found significant effects of exclusion on subjective affective state (inclusion: mean 6.76 (SD:1.37); exclusion: mean 6.5 (SD: 1.51); t(87) = −3.813,p < 0.001) and anger (inclusion: mean 2.45 (SD: 1.23); exclusion: mean 3.18 (SD: 1.75); t(48) = 4.762;p < 0.001; Fig. 2).

### Mood Change

PANAS values differed significantly for positive and negative affective state (F(1,85) = 1106.73; p < 0.001) and measurement time point (with lower values in both; F(1,85) = 33.34;p < 0.001). The interaction between the two factors was also significant (F(1,85) = 13.31,p < 0.001, Fig. 2).

### Integration of brain and behavior

The repeated measures ANCOVA for exclusion was still significant for subjective affective state (F(1,84) = 6.17;p = 0.015). The connectivity change value of the dACC cluster also showed a significant association with the exclusion effect in this model (F(1,84) = 3.94; p = 0.05). Other clusters did not reach significance, neither did any interaction (nor did they show a trend at p < 0.1). For anger the main effect of exclusion was also significant (F(1,45) = 14.15;p < 0.001). None of the covariates showed a significant association or interaction. Nevertheless, the interaction with the IFG cluster was at trend level (F(1,45) = 3.55;p = 0.066, Fig. 2).

Entering connectivity values as covariates in the analysis of mood change did not change significance, nor did any of the covariates or interactions reach significance or trend level.

## Discussion

In the present study, we investigated the influence of social stress induced by social exclusion on stability of large-scale brain networks, by comparing resting-state fMRI before and after the cyberball task in a large sample of healthy participants. With respect to behavioral effects, the present results corroborate previous findings: the cyberball task leads to lower positive affect, elevated anger during exclusion periods compared to inclusion^26^,^36^. Positive and negative mood state dropped over time, which is a stress-like effect, that has been shown in previous studies^37^. Previous studies clearly demonstrated that exclusion in a social context is experienced as distressing, as it threatens fundamental needs such as sense of belonging and meaningful existence^14^,^16^,^20^. Aside from these behavioral findings, we were able to demonstrate that the functional connectivity of the resting brain is significantly modulated following the cyberball task. This constitutes the first experimental study demonstrating modulatory effect of social stress induced by social exclusion on resting-state functional connectivity.

While SN and ECN connectivity stay stable, the DMN selectively shows an elevated connectivity with hubs of the SN^38^, higher order visual areas that covary with subjective experience in various tasks^39^ and sensori-motor areas. A possible interpretation of these changes as a stress-induced reconfiguration of the DMN towards a more attentive, vigilant state involving core hubs of salience processing, higher order visual and sensorimotor areas in the aftermath of stress.

These results are based on a regression, which depicts uniquely explained variance of each of the three input networks. Therefore, we can argue that SN and ECN remain unchanged by the task (or experimental session) but that the DMN selectively displays increased connectivity to core hubs of the SN, visual processing areas and sensori-motor areas. Based on the present results, a plausible suggestion is that the DMN shifts its processing mode from spontaneous, self-referential mental activity and internal mentation in the absence of external demands^40^,^41^,^42^, towards a more vigilant and attentive mode, sub-served by key nodes of the SN and visual as well as motor areas.

Given this assumption, our results corroborate and extend the theoretical framework outlined by Hermans and colleagues^1^. Specifically, our results are in line with the authors’ earlier proposal that the adaptation of the default mode of brain function is primarily mediated by a reallocation of neural resources, which is driven by noradrenergic bottom-up modulation triggered by the stressor^13^. As the interplay between the dACC and the anterior insula is commonly associated to modulation of subcortical structures via noradrenergic pathways, our results could very well be explained via this association (cf 1). Our results however extend previous findings by indicating that also a relative, social stressor such as the cyberball task potentially induces the proposed reallocation of neural resources. Interestingly, DMN connectivity is mainly elevated in regions which have been shown to be responsive to social exclusion^15^,^24^,^26^. Potentially, this is a result of ongoing evaluation of the stressful situation participants were exposed to during the cyberball task. This further supports the link between social stress and reallocation of network resources.

Nevertheless, we predicted social stress effects changes in the ECN and SN itself. It should be noted that connectivity changes within these two large-scale brain networks occur immediately after stress onset, and that connectivity gradually returns to a pre-stress level afterwards^1^. The time frame outlined by Hermans and colleagues predicts that stress-induced changes SN and ECN return to pre-stress level roughly one hour after stress onset, representing approximately the same amount of time that passed between paradigm onset and acquisition of the second resting-state measurement in the present study. Thus, it could be the case that this time delay prevented us from detecting changes in SN and ECN. Connectivity patterns of these networks might have already normalized or were in the course of normalizing, which may reflect the aftermath of stress, and in this, effects may potentially be observable in DMN in the sense of a more long term preparatory adaptation.

Such a proposed adaptation after prolonged exposure to a stressor may be evolutionary useful: The DMN connects more strongly to regions processing salience, attention and negative affect. This adaptive connectivity may prepare the individual for reorienting attention (to potential stressors or threats), detect salient events in the environment, mobilize energy (to initiate fight or flight responses if necessary), and take rapid as well as unpremeditated actions. In the aftermath of a stressful situation, these processes might be highly beneficial.

Enhanced DMN connectivity, as observed in the current study may depict a shift towards a more salient mode of brain function in response to social stress. Adaptive responses of the DMN may represent a profound change induced by the cyberball task, reflecting more long-term adaption in reaction to stress. Such long term-adaptations in resting-state functional connectivity patterns have been observed for example in patients suffering from post-traumatic stress disorder (PTSD). These patients show exactly this pattern, namely elevated interconnectivity between the DMN and SN^43^, which may reflect a chronified ‘alert default state’ of the brain.

Specifically, the dACC may be a crucial hub for such processes as it integrates interoceptive autonomic processing^44^,^45^, different forms of salience detection^44^,^46^,^47^, error detection and conflict monitoring^48^,^49^, social pain and negative affect processing^50^, and coordinating preparatory and stimulus-driven attentional activity^51^,^52^. Furthermore, it has been demonstrated that the dACC is densely and reciprocally connected to the cholinergic and noradrenergic subcortical systems responsible for the cognitive control of arousal^53^,^54^,^55^,^56^. In conclusion, dACC might be responsible for controlling subcortical structures providing noradrenergic bottom-up activity, which would lead to an increased default resting alertness in the aftermath of stress. Similarly, the anterior insula is well known for its role in regulatory and integrative attentional functions^56^,^57^,^58^, detection of novel and salient stimuli^59^,^60^, and subjective awareness^61^.

Obviously, by not having a control condition and by only observing weak significant or trending associations between behavior and change in brain connectivity, we cannot be certain about the specificity of this effect to the social stressor. It might simply be time and unspecific cognitive effects that drive the connectivity change in the DMN from the first to the second time point. Indeed, there is evidence that physiologically relevant large-scale brain networks are consistent over time^62^,^63^,^64^,^65^,^66^. These studies convincingly demonstrated that within-subjects, across scan stability and spatial consistency of large-scale brain networks are high for healthy participants. High test-retest reliability has also been shown for specific indices of resting-state functional connectivity, such as centrality measures^67^, amplitude measures of spontaneous low-frequency fluctuations^68^, and regional homogeneity^69^. Furthermore, several studies demonstrated that using independent component analysis (ICA) to analyze resting-state fMRI data results in high to excellent reliability of functional connectivity, particularly within heteromodal associative large-scale brain networks such as the DMN^70^,^71^,^72^,^73^. Hence, within-subject, across scan stability of large-scale brain networks, including those networks examined here, is remarkably high for healthy volunteers.

Nevertheless, we cannot completely exclude the possibility that changes in resting-state functional connectivity might occur without any intervention or task in between, as we have not directly examined the stability of the large-scale brain networks without a stress-inducing task in between. Indeed, as the above mentioned studies render mere time effects on large-scale brain networks highly unlikely, an interesting alternative explanation for the observed changes in DMN connectivity may be that the task induced some kind of cognitive fatigue or mental exhaustion. These processes have been shown to elicit (possibly compensatory) increases in connectivity of DMN to frontal midline structures^74^,^75^ and would also explain decrease in positive and negative affect (although these behavioral changes were not significantly associated to connectivity change). Future studies, employing appropriate within- or between-subjects experimental designs testing the stress versus cognitive fatigue effect on the DMN seem necessary to resolve this issue.

Similarly, we did not focus on gender as a potential moderating factor in these analyses. A recent meta-analysis of 120 cyberball studies found no effect of gender on social exclusion^76^. The authors demonstrated that (i) the average ostracism effect induced by the cyberball task generalizes across several sampling characteristics (i.e., gender, age), and (ii) that the proportion of male and female participants did not predict the mean effect size. Thus, although potentially interesting in the context of the effect of male or female only excluding group, we did not modulate this factor and thus did not include it into our analysis.

## Conclusions

The present study demonstrates that the cyberball task may induce changes in affective processing and also resting-state functional connectivity of the DMN. In the aftermath of social stress, the default mode of the brain has to adapt and is shifted towards a state of increased vigilance in order to prepare the individual for reorienting attention, detecting salient stimuli and mobilizing energy to initiate flight responses if necessary. Our results can be interpreted as a neural substrate of this more vigilant and attentive state, manifesting at the systems level in the form of increased connectivity of the DMN with key areas of the SN and other brain areas involved in sensorimotor and visual processes necessary to enable rapid adaptive responses to (upcoming) environmental changes and stressors. Our results are in line with a recent proposal of stress related large-scale brain dynamics^1^ and provide first experimental evidence for the putative modulatory influence of social stress on the stability of large-scale brain networks in the aftermath of stress. If further substantiated, this finding may also have implications for psychopathological alterations of the stress response in a social context as in many psychiatric disorders.

## Additional Information

**How to cite this article**: Clemens, B. *et al*. Alerted default mode: functional connectivity changes in the aftermath of social stress. *Sci. Rep.* **7**, 40180; doi: 10.1038/srep40180 (2017).

**Publisher's note:** Springer Nature remains neutral with regard to jurisdictional claims in published maps and institutional affiliations.

## Figures and Tables

**Table 1:** Overview of resting-state fMRI results.

Anatomical Region	BA	X	Y	Z	*p* - value	No. of voxels
Post-Cyberball > Pre-Cyberball DMN						
L dACC	32	−6	28	32	0.04	23
R post-central gyrus	3	50	−14	26	0.03	21
R IFG/anterior insula	45/47	42	36	8	0.033	21
R pre-SMA/premotor cortex	6	22	8	50	0.027	89
R IFG/anterior insula	44	48	12	12	0.011	225
R secondary visual cortex (V2)	18	4	−62	18	0.028	245
R extrastriate visual cortex (V3a)	19	18	−74	26	0.039	40

All x, y, and z values represent coordinates according to the MNI coordinate system (ICBM 152). Statistical values correspond to the *p*-values of the peak voxel within each anatomical region. Thresholding and correction of multiple comparisons was achieved using the threshold-free cluster enhancement (TFCE) method (*p* < 0.05). (*BA* = *Brodmann area; dACC* = *dorsal anterior cingulate cortex; DMN* = *default mode network; IFG* = *inferior frontal gyrus; L* = *left hemisphere; R* = *right hemisphere; SMA* = *supplementary motor area*).
